# “Villains” Turning Good: Antimycin A and Rotenone, Mitochondrial Respiratory Chain Inhibitors, Protect H9c2 Cardiac Cells Against Insults Triggering the Intrinsic Apoptotic Pathway

**DOI:** 10.3390/ijms26062435

**Published:** 2025-03-08

**Authors:** Kyriaki Zikaki, Eleni Kiachaki, Catherine Gaitanaki, Ioanna-Katerina Aggeli

**Affiliations:** Section of Animal and Human Physiology, Faculty of Biology, School of Science, National and Kapodistrian University of Athens, University Campus, Ilissia, 15784 Athens, Greece; kirkizikaki@gmail.com (K.Z.); eleni.kiachaki@gmail.com (E.K.); cgaitan@biol.uoa.gr (C.G.)

**Keywords:** mitochondria, reactive oxygen species, electron transport chain, antimycin A, rotenone, calcium signaling, oxidative stress, integrated stress response

## Abstract

Mitochondria are the powerhouses of cells, also involved in ROS (reactive oxygen species) generation and cellular death regulation. Thus, several diseases are associated with mitochondrial impairment, including cardiovascular disorders (CVDs). Since CVDs are currently the leading cause of death worldwide, it is very important to evaluate targeting mitochondrial effectors in clinical treatment protocols. Hence, in the present study, antimycin A and rotenone, established inhibitors of the mitochondrial electron transfer chain, were shown to halt apoptotic death induced by curcumin (50 μM) and sorbitol (0.5 M), in H9c2 cardiac cells. In particular, immunoblotting analysis revealed that they totally abolished PARP [poly(ADP-ribose) polymerase] proteolysis, under these conditions. This finding was accompanied by an enhancement of cell viability, recovery of mitochondria networks’ integrity, suppression of cytochrome c release into the cytoplasm, and reversal of chromatin condensation. Chelating extracellular calcium (with EGTA) further enhanced the beneficial impact of antimycin A and rotenone on curcumin- or sorbitol-treated H9c2 cells viability. Of interest, the phosphorylation of eIF2α, indicative of the onset of the pro-survival Integrated Stress Response (IRS), was sustained under these conditions. Overall, our data highlight the anti-apoptotic effect of these compounds, unmasking their potential as mediators in novel therapeutic interventions against mitochondria-associated cardiac dysfunction.

## 1. Introduction

The preservation of redox equilibrium is a constant challenge for aerobic organisms, with oxidative imbalances undermining their wellbeing. Reactive oxygen species (ROS) play an essential role as signaling molecules, regulating cell physiology and function. Nonetheless, if the cellular antioxidative mechanisms fail to maintain ROS in balance, excessive amounts of the latter accumulate, causing the disruption of signal transduction pathways and the impairment of proteins, lipids, and nucleic acids, via the oxidation of their macromolecular structure [1]. As anticipated, studies have established that the resulting oxidative stress is involved in the pathophysiology of a plethora of diseases affecting the cardiovascular system, along with the nervous, the muscular, the immune system, etc. [2]. Mitochondria are largely responsible for cellular ROS generation, along with NADPH oxidases (Noxs) and other enzymatic moieties (i.e., xanthine oxidoreductase, cyclooxygenase, etc.) [3]. In mitochondria, ROS are generated as byproducts, on the course of oxidative phosphorylation reactions, that are carried out by protein complexes located in the inner mitochondrial membrane. These supercomplexes constituting the mitochondrial electron transport chain (ETC) include four respiratory cytochrome-based enzymatic complexes (complexes I, II, III, and IV), which generate a proton gradient across the inner mitochondrial membrane, and a fifth one (complex V), the ATP synthase, that, ultimately, converts ADP to ATP, providing cells with their “energy currency” [4].

ROS are produced in the ETC due to electron leakage, as electrons are transferred from reduced forms of nicotinamide adenine dinucleotide (NADH) and flavine-adenine dinucleotide (FADH2) to oxygen, as well as when electrons deviate and swerve at complexes I and III. Consistently, mitochondrial complexes I and III have been identified as the major sources of superoxide radicals in mitochondria, with complex I releasing them solely in the matrix, while complex III also releases them into the intermembrane mitochondrial space [5]. Given the significance of untangling the regulatory mechanisms of these processes, in an effort to alleviate their detrimental impact as inducers of oxidative stress, researchers have resorted to the use of inhibitors of the respiratory chain. The most widely applied and extensively evaluated are antimycin A and rotenone [6]. Antimycin A, derived from *Streptomyces* sp., has antibiotic properties and inhibits complex III of the ETC, by irreversibly binding to the Qi subunit in the core of cytochrome B. It is registered as a pesticide, applied to terminate the presence of invasive or non-native species, promoting the prevalence of indigenous ones [7]. Through its use as a piscicide for fish cultures, antimycin A may present toxicity in humans by the consumption of plants or fish. On the other hand, rotenone, extracted from the roots and stems of *Lonchocarpus* and *Derris* sp. plants, is an isoflavone that reversibly inhibits mitochondrial complex I and has been widely used as an insecticide [8]. While rotenone has been characterized as toxic, it has also been found to exert a beneficial effect in several disease experimental models [9,10,11]. Intriguingly, so as to bring out their therapeutic value, it should be noted that both antimycin A and rotenone have proven, among other oxidative phosphorylation inhibitors, their efficacy to eliminate transformed cells, in cases of cancer chemotherapy resistance [12]. Therefore, since “mitochondrial medicine” has arisen as a promising scientific field, deciphering aspects of mitochondrial function could contribute to the development of therapeutic strategies against an array of disorders, including cardiovascular diseases.

The heart is in need of a constant provision of high levels of energy to accommodate for uninterrupted contractility. This is achieved by using different substrates, depending on the phase of cardiac development or occurrence of pathological conditions. In fact, while the heart primarily depends on aerobic glycolysis and lactate oxidation during fetal development, in adulthood, it relies on oxidative metabolism, supported by a remarkable increase in mitochondrial volume mass and with fatty acids becoming the predominant fuel. Thus, cardiac myocytes are totally dependent on mitochondrial bioenergetic output, with mitochondria occupying ~30–40% of their volume and generating ~90% of the ATP required for their function. In addition, mitochondria play a crucial role in the preservation of cardiac cell homeostasis, due to their vital role in redox equilibrium, with oxidative stress linked to heart failure, ischemic heart disease, and cardiac hypertrophy [13]. They are also involved in cardiac contractile function through their involvement in calcium handling. In particular, they absorb Ca^2+^ as a response to cytosolic calcium increases, mainly through the mitochondrial calcium uniporter (MCU), a transporter located in the inner mitochondrial membrane [14,15]. Paradoxically, mitochondria are essential not only for powering life, but also for regulating cell death. Indeed, these organelles orchestrate several modes of cellular death including apoptosis, pyroptosis, necroptosis, autophagy, ferroptosis, etc. [16]. With cardiac myocytes being terminally differentiated and, thus, having a minimal potential for regeneration, any cell loss can result in myocardial impairment and dysfunction.

Apoptosis, featuring chromatin condensation, membrane blebbing, DNA breakdown, and the formation of apoptotic bodies, is α programmed cell death modality executed via an extrinsic or intrinsic pathway [17]. Mitochondria are related to the endogenous pathway, with mitochondrial outer membrane permeabilization (MOMP) paving the way to the ensuing death. Hence, upon activation, pro-apoptotic members of the Bcl-2 family of proteins (primarily Bax and Bak) shuttle between the mitochondria and cytoplasm, ultimately inducing the formation of lipidic pores in the mitochondrial outer membrane. Next, the effectors released, initiate proteolytic activity, conferring the degradation of cytosolic as well as nuclear targets [18]. Cytochrome c is a central molecule in this process, which, once released, associates with other mediators to form the apoptosome [19]. A cascade of consecutive proteolytic cleavage and subsequent activation of caspases is, next, triggered, with their effector members cleaving and activating, in turn, a broad range of targets [20]. The proteolytic processing of PARP-1 is an established hallmark of apoptosis [21]. PARP cleavage into a 89 kDa or smaller-sized fragments impairs the ability of this enzyme to act as a sensor of DNA strand breaks and to contribute to their repair [17]. Interestingly, several reports have marked an unanticipated reversal of late-stage apoptosis, as an important mechanism of cell rescue under stressful conditions. In particular, in their review, Tang and Tang elaborate on “anastasis”, a natural cell recovery process that “rescues cells from the brink of death” [22].

In previous reports, we had already shown that curcumin [23] as well as hyperosmotic stress conferred by sorbitol [24,25] both triggered the intrinsic apoptotic pathway. Hence, in the present study, we made an effort to investigate the effect of antimycin A and rotenone on the cellular responses triggered under these severe stress conditions in H9c2 cardiac cells. Noteworthy, herein, antimycin A as well as rotenone were found to effectively hinder the induced PARP proteolysis, reverse chromatin condensation, and restore mitochondrial morphology and integrity under the aforementioned conditions, resulting in an enhancement in cell survival. The inhibition of calcium uptake was determined to also play a crucial role in preserving cell homeostasis. Furthermore, the Integrated Stress Response (IRS), evidenced by eIF2 alpha phosphorylation [26] which was elicited in order to offer protection to H9c2 cells against curcumin- and sorbitol-induced deleterious repercussions, was found to be sustained in the presence of antimycin A as well as rotenone. Therefore, both mitochondrial ETC inhibitors were found to exert a salutary effect via reversing the pro-death outcome initially observed under the conditions examined. Further delineating the mechanisms of interplay between mitochondrial effectors and cellular responses triggered by stressful cues is of crucial significance and may contribute to new therapeutic approaches against mitochondria-associated diseases, affecting organs with high-energy requirements including: the myocardium, brain, and skeletal muscles [27].

## 2. Results

### 2.1. Curcumin [50 μM] Confers an Overwhelming Apoptotic Insult in H9c2 Cells

**Figure 1:**
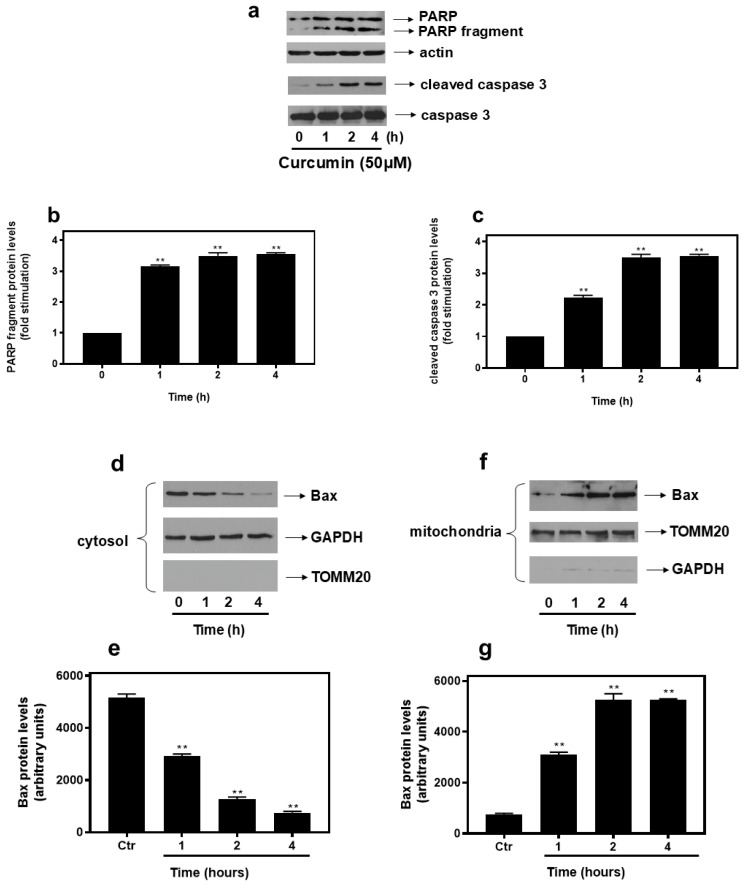
Time-dependent pattern of curcumin–induced PARP proteolysis (**a**), caspase 3 cleavage (**a**), and Bax translocation (**d**,**f**) from cytosol to mitochondria, in H9c2 cardiac cells. H9c2 cells were exposed to 50 μM curcumin for the times indicated. Protein extracts (40 μg/lane) were subjected to SDS-PAGE and immunoblotted with antibodies for PARP (full length and fragments: upper panel), for caspase 3 (fragment and uncleaved: 3rd and bottom panels, respectively), for total levels of Bax (upper panels), for actin levels (**a**: 2nd panel), for GAPDH levels (**d**: middle panel, **f**: bottom panel), and for TOMM20 levels (**d**: bottom panel, **f**: middle panel). Western blots presented are representative of at least three independent experiments with overlapping results. Immunoreactive bands were quantified by scanning densitometry and plotted (respective graphs: (**b**,**c**,**e**,**g**)). Results are means ± SEM for at least three independent experiments. ** *p* < 0.01 compared to control values.

Curcumin has been associated with mitochondrial dysfunction and oxidative stress. We have previously shown that, at 20 μM, curcumin triggers an oxidative-stress-mediated pro-apoptotic effect on H9c2 cells, moderately reducing the cells’ viability [23]. Hence, in the present study, we decided to challenge H9c2 cells with a much higher concentration of this natural polyphenol [50 μM], in order to elucidate the respective mechanisms triggered, severely affecting H9c2 cells’ survival. Consistently, apoptosis was found to be significantly induced as early as 1 h after the treatment of cells with 50 μM curcumin, evidenced by the cleavage of PARP (3.15 ± 0.3-fold relative to control, *p* < 0.01) (Figure 1a upper panel and b) as well as by the proteolysis of caspase 3 (3.59 ± 0.15-fold relative to control, *p* < 0.01) (Figure 1a 3rd panel from top and c). Equal protein loading was verified by the immunodetection of actin (Figure 1a 2nd panel from top). With Bax known to translocate to the mitochondria upon activation, promoting mitochondrial outer membrane permeabilization, we next examined its time-dependent expression in the cytosolic and the mitochondrial fractions of H9c2 cells, treated under the aforementioned conditions. In agreement with PARP and caspase 3 fragmentation patterns, treatment with curcumin led to a progressive reduction in the Bax presence in the cytosol (~43% after 1 h relative to control, *p* < 0.01) (Figure 1d upper panel and e), while it was concomitantly gradually enhanced (~3-fold relative to control, *p* < 0.01) in the mitochondrial enriched fraction (Figure 1f upper panel and g). Equal protein loading was verified by the immunodetection of GAPDH (glyceraldehyde-3-phosphate dehydrogenase), an established cytosolic marker (Figure 1d middle panel) and TOMM20, a routinely used mitochondrial marker (Figure 1f middle panel). So as to validate the isolation and verify the enrichment of the mitochondrial and cytosolic fractions, membranes were also probed with antibodies against GAPDH and TOMM20, respectively (Figure 1d,f bottom panels, respectively).

### 2.2. MAPK Inhibitors and Anti-Oxidants Cannot Alleviate Curcumin-Induced Apoptosis in H9c2 Cells

Since all MAPK (Mitogen-activated protein kinase) superfamily members have been reported to be activated by curcumin [28], we next made an effort to study their potential involvement in the detrimental apoptotic insult, after the treatment with curcumin (50 μM). To this end, we used selective pharmacological inhibitors: U0126 (10 μM) for ERKs (Extracellular signal-regulated kinases), SB203580 (10 μM) for p38-MAPK, and AS601245 (2.5 μM) for JNKs (c-Jun N-terminal protein kinases), respectively. None of the inhibitors tested affected curcumin-induced PARP proteolysis (Supplementary Figure S1a,b: upper panels). Interestingly, although treatment with curcumin has been shown to promote oxidative stress, which, in turn, mediates the apoptotic insult observed [23], the PARP fragmentation in H9c2 cells treated with 50 μM curcumin for 1 h remained unchanged in the presence of SOD [30 U/mL] and NAC [10 mM], established antioxidants (Figure S1c upper panel). Equal protein loading was once again verified by the immunodetection of actin (Figure S1 bottom panels). This result indicated the stimulation of a different signal transduction mechanism under these conditions, rendering the identification of the effectors involved compelling.

### 2.3. Antimycin A Completely Abolishes Curcumin-Induced PARP Proteolysis in H9c2 Cells, with HOE642 and Apocynin Exerting No Inhibitory Effect

**Figure 2:**
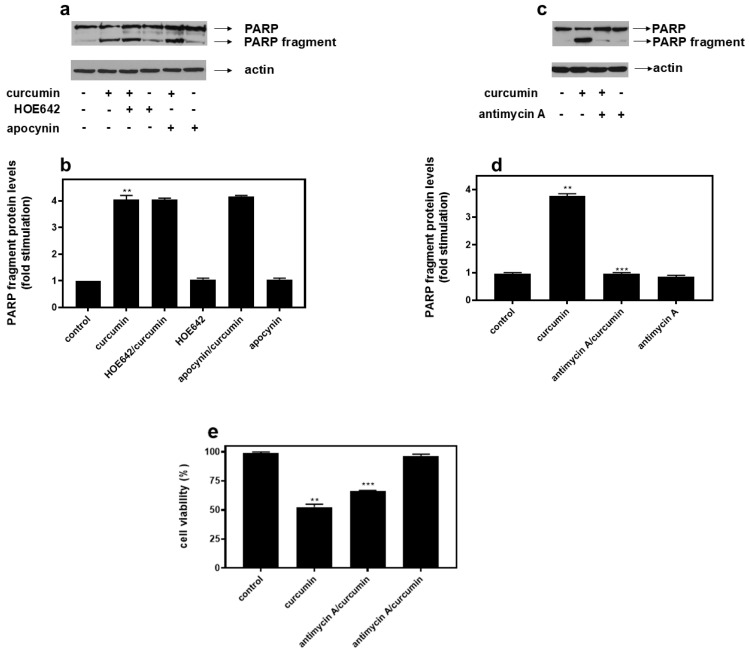
While HOE642, a specific inhibitor of NHE-1, as well as apocynin, an inhibitor of Nox, do not affect curcumin–induced PARP proteolysis (**a**), the latter is abolished by antimycin A (**c**). H9c2 cells were left untreated or were incubated with the inhibitors alone or with the inhibitors followed by exposure to 50 μM curcumin in the presence of the inhibitors. Cell extracts (40 μg/lane) were subjected to SDS-PAGE and immunoblotted with antibodies that detect PARP (full length and fragments—upper panels), or total levels of actin (bottom panels). Western blots are representative of at least three independent experiments with overlapping results. Immunoreactive bands were quantified by scanning densitometry and plotted (respective graphs: (**b**) and (**d**), respectively). Results are means ± SEM for at least three independent experiments. ** *p* < 0.01 compared to control values; *** *p* < 0.01 compared to curcumin-treated cells in the absence of the inhibitors. (**e**) Assessment of H9c2 (%) cell viability by trypan blue exclusion assay. H9c2 cells were left untreated (control), treated with curcumin, with antimycin A alone, or with antimycin A followed by exposure to curcumin in the presence of antimycin A (antimycin A/curcumin). All experiments were performed in triplicate. Values are means ± SEM for three independent experiments. ** *p* < 0.05 compared to control values; *** *p* < 0.01 compared to curcumin-treated cells in the absence of antimycin A.

Taking into consideration that Na^+^/H^+^ exchanger-1 (NHE-1) and NADPH oxidase (Nox) constitute enzymes of fundamental significance in ROS generation, as well as their direct association with the mitochondrial ETC, we subsequently investigated the effect of specific inhibitors of their activity on curcumin-induced PARP proteolysis. In particular, H9c2 cells were left untreated or were incubated with the inhibitors alone, or with the inhibitors followed by exposure to curcumin 50 μM for 1 h. Thus, in the presence of HOE642 (5 μM), a specific inhibitor of NHE-1, as well as of apocynin (50 μM), known to inhibit Nox, no modulation of the levels of curcumin-induced PARP fragment was observed (Figure 2a upper panel and b). Equal protein loading was once again verified by the immunodetection of actin (Figure 2a bottom panel). Pursuing the effort to determine the effectors mediating the deleterious effect of curcumin, we next pre-incubated H9c2 with antimycin A (0.1 μM), which disrupts the electron flow through the mitochondrial chain via inhibiting complex III. Intriguingly, in the presence of antimycin A, curcumin-induced PARP cleavage was completely abrogated (Figure 2c upper panel and d). Of note, this protective effect of antimycin A correlated with a statistically significant augmentation in H9c2 cell viability, observed after performing a trypan blue exclusion analysis in cells pre-incubated with antimycin A and subsequently exposed to 50 μM curcumin for 1 h (Figure 2e).

Next, after treating H9c2 cells with 50 μM curcumin for 1 h and staining them with Hoechst 3325, an upregulation of nuclear fluorescence and chromatin condensation levels were observed, compared to untreated cells, further indicating the occurrence of apoptosis (Figure S2: curcumin vs. control panel). Antimycin A [0.1 μM] inhibited curcumin-induced DNA condensation, as evidenced in Figure S2 (antimycin A/curcumin vs. curcumin panel).

**Figure 3:**
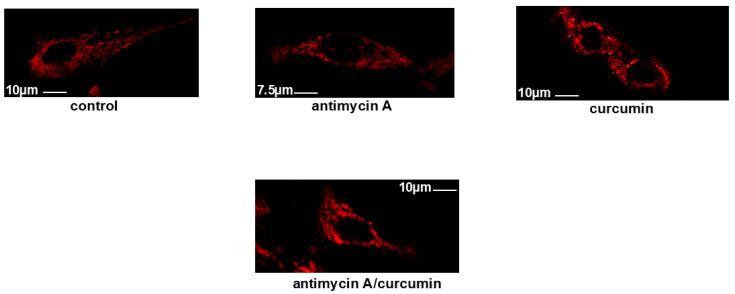
Antimycin A preserves mitochondrial integrity. Using Mitotracker Red CMXRos, the mitochondrial morphology of H9c2 cells was studied. Mitochondria in untreated H9c2 cells (**control**) as well as in antimycin A-treated cells (**antimycin A**) appeared to be intact, forming a well-shaped tubular network. On the contrary, treatment with curcumin (**curcumin**) rendered mitochondrial morphology punctate, indicative of their distorted function. In H9c2 cells pre-incubated with antimycin A and subsequently exposed to curcumin in the presence of the inhibitor (**antimycin A**/**curcumin panel**), a tubular well-preserved mitochondrial network was once more observed.

Accordingly, confocal images of cells stained with 100 nM MitoTracker Red CMXRos revealed that, while untreated H9c2 cells (**control**) as well as antimycin A-treated cells (**antimycin A**) appear to have intact mitochondrial networks, treatment with curcumin (**curcumin**) rendered the mitochondrial morphology fragmented and punctate. Pre-incubation with antimycin A once again reversed curcumin’s impact, restoring the mitochondrial morphology (**antimycin A/curcumin**) (Figure 3).

### 2.4. Role of Calcium in the Beneficial Effect of Antimycin A Against Curcumin-Induced Cellular Death in H9c2 Cells

**Figure 4:**
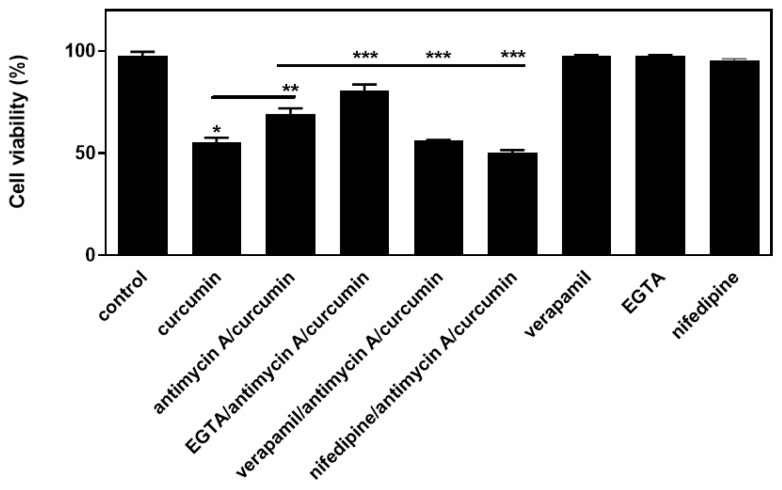
Antimycin A exerts a salutary effect on H9c2 cell viability which appears to be associated with extracellular calcium, as well as L-type calcium channels. Measuring H9c2 (%) cell viability by trypan blue exclusion assay, cells were left untreated (control), treated with curcumin (curcumin), or treated with antimycin A followed by exposure to curcumin in the presence of antimycin A (antimycin A/curcumin), or exposed to calcium-related compounds (EGTA, verapamil, and nifedipine) before being treated with antimycin A and curcumin. All experiments were performed in triplicate. Values are means ± SEM for three independent experiments. * *p* < 0.05 compared to control values; ** *p* < 0.01 compared to curcumin-treated cells; *** *p* < 0.01 compared to cells pre-treated with antimycin A and subsequently exposed to curcumin, in the presence of antimycin A.

Given that mitochondria play a significant role in modulating intracellular calcium homeostasis, an effort was next made to probe into the potential role of calcium-related effectors in the observed salutary impact of antimycin A. In this context, the effect of a number of compounds on cellular viability was assessed. In particular, we used ethylene glycol tetraacetic acid (EGTA-1 mM), a cell-impermeable chelating agent with a high affinity for calcium ions, as well as nifedipine (1 μM) and verapamil (1 μM), which both block voltage-dependent L-type calcium channels, thereby inhibiting calcium influx. Thus, after performing the trypan blue exclusion assay, we found that, in the presence of EGTA, the reduction conferred by curcumin in H9c2 viability, partly alleviated by antimycin A, was further overturned (by 17 ± 1.4%, *p* < 0.01 compared to antimycin A/curcumin-treated cells). Of note, the salutary effect of antimycin A was reversed by both verapamil (by 18.8 ± 0.6%, *p* < 0.01 compared to antimycin A/curcumin-treated cells) and nifedipine (by 27.5 ± 0.9%, *p* < 0.01 compared to antimycin A/curcumin-treated cells) (Figure 4). These findings demonstrate that chelating extracellular calcium and, thus, totally blocking calcium influx appears to have a beneficial effect on H9c2 cell viability. On the other hand, the blockade of the L-type voltage-dependent calcium channels aggravated the detrimental impact of curcumin, eliminating the beneficial effect conferred by antimycin A.

### 2.5. Hindering Activity of Mitochondrial Complex I with Rotenone Equally Overturns Curcumin-Induced PARP Fragmentation in H9c2 Cells

**Figure 5:**
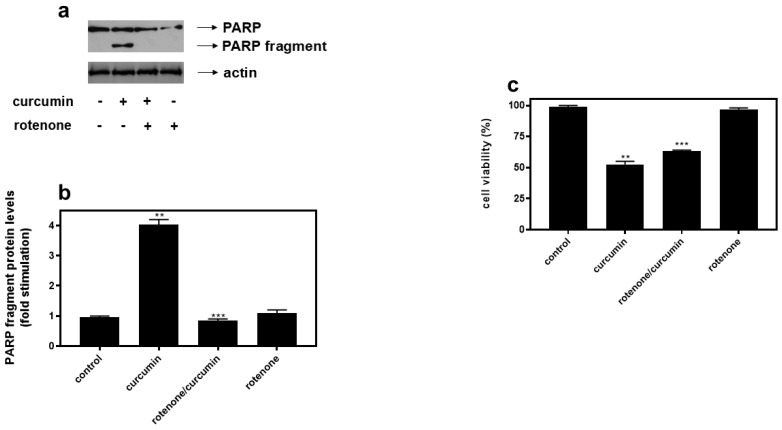
Rotenone (0.1 μM) completely suppresses curcumin–induced PARP cleavage. H9c2 cells were left untreated (control) or were incubated with rotenone alone (rotenone), or with rotenone followed by exposure to 50 μM curcumin in the presence of the inhibitor (rotenone/curcumin). (**a**) Cell extracts (40 μg/lane) were subjected to SDS-PAGE and immunoblotted with antibodies that detect PARP (full length and fragments—upper panel), as well as total levels of actin (bottom panel). Western blots are representative of at least three independent experiments with overlapping results. Immunoreactive bands were quantified by scanning densitometry and plotted (**b**). Results are means ± SEM for at least three independent experiments. ** *p* < 0.01 compared to control values; *** *p* < 0.01 compared to curcumin-treated cells in the absence of the inhibitor. (**c**) Measurement of H9c2 (%) cell viability by trypan blue exclusion assay. H9c2 cells were left untreated (control), treated with curcumin (curcumin), with the inhibitor compound alone (rotenone), or with the inhibitor followed by exposure to curcumin in the presence of rotenone (rotenone/curcumin). All experiments were performed in triplicate. Values are means ± SEM for three independent experiments. ** *p* < 0.05 compared to control values; *** *p* < 0.01 compared to curcumin-treated cells in the absence of rotenone.

With ROS being primarily generated by complex III and I of the mitochondrial respiratory chain, we subsequently probed into the effect of inhibiting complex I by rotenone (0.1 μM). Hence, curcumin-induced PARP proteolysis was completely inhibited when H9c2 cells were pre-treated with rotenone (0.1 μM) (Figure 5a upper panel and b). This rotenone-conferred abolishment of the apoptosis triggered by curcumin correlated with a statistically significant amelioration in the survival of H9c2 cells (by approximately 20 ± 0.4%, *p* < 0.01 compared to curcumin-treated cells), as shown in Figure 5c.

### 2.6. Antimycin A and Rotenone Inhibit Apoptosis Induced by Sorbitol in H9c2 Cells

In order to determine whether the beneficial anti-apoptotic effect of inhibiting complex I and III of the mitochondrial respiratory chain was specific for H9c2 cells exposed to curcumin (50 μM), the effect of these inhibitors was also studied after the exposure of cells to hyperosmotic stress induced by 0.5 M sorbitol.

**Figure 6:**
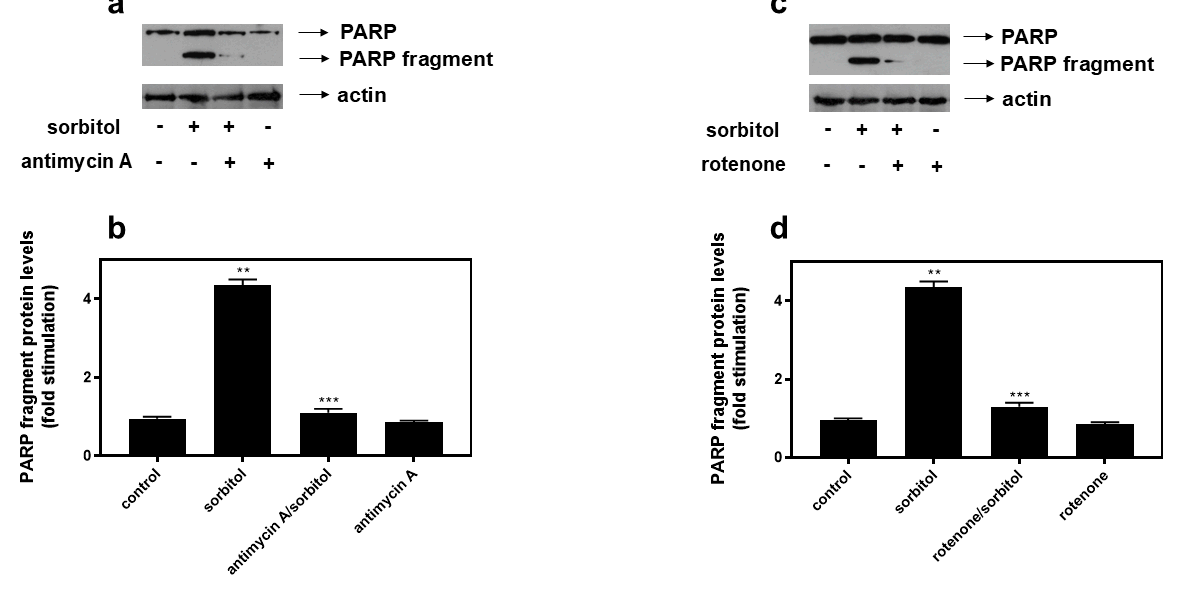
Antimycin A (0.1 μM) as well as rotenone (0.1 μM) both equally abolish sorbitol-induced PARP fragmentation. H9c2 cells were left untreated (control) or were incubated with antimycin A (antimycin A) or rotenone alone (rotenone), or with the inhibitors followed by exposure to sorbitol (0.5 M) in the presence of the inhibitors (antimycin A/sorbitol or rotenone/sorbitol). (**a**,**c**) Cell extracts (40 μg/lane) were subjected to SDS-PAGE and immunoblotted with antibodies that detect PARP (full length and fragments—upper panels), as well as total levels of actin (bottom panels). Western blots are representative of at least three independent experiments with overlapping results. Immunoreactive bands were quantified by scanning densitometry and plotted ((**b**,**d**): respective graphs). Results are means ± SEM for at least three independent experiments. ** *p* < 0.01 compared to control values; *** *p* < 0.01 compared to sorbitol-treated cells in the absence of the inhibitor.

As illustrated in Figure 6a,c (upper panels), antimycin A and rotenone both totally suppressed PARP proteolysis, and, hence, apoptosis, triggered in H9c2 cells treated for 1 h with 0.5 M sorbitol, indicating that the beneficial impact of these compounds is not stimulus-specific. This pro-survival effect rather appears to be closely associated with particular effectors of the mitochondrial ETC function, indispensable for the occurrence of apoptosis.

**Figure 7:**
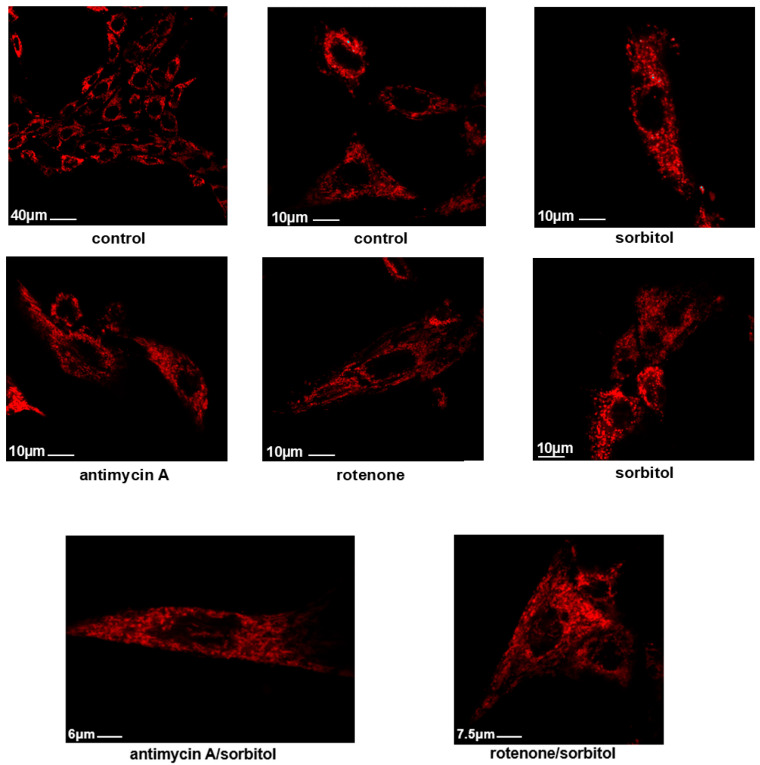
Antimycin A and rotenone preserve mitochondrial integrity under conditions of hyperosmotic stress. Using Mitotracker Red CMXRos, the mitochondrial morphology of H9c2 cells was studied. Mitochondria in untreated H9c2 cells (**control**), and in antimycin A—as well as in rotenone-treated cells (**antimycin A** and **rotenone**, respectively) appeared to be intact. On the other hand, treatment with sorbitol rendered mitochondrial morphology punctate and fragmented. In H9c2 cells pre-incubated with either antimycin A or rotenone and subsequently exposed to sorbitol in the presence of either inhibitor, a tubular well-preserved mitochondrial network was once more observed (**antimycin A/sorbitol** and **rotenone/sorbitol**, respectively). Cells were visualized under a Zeiss Axioplan fluorescence microscope (bar scale ranging from 6–40 μM, as indicated).

Looking into the mitochondrial morphology of H9c2 cells pre-incubated with antimycin A or rotenone and subsequently exposed to sorbitol, in the presence of the aforementioned compounds, further corroborated the beneficial effect of the latter. Using Mitotracker Red CMXRos revealed that mitochondria in untreated H9c2 cells (**control**) as well as in antimycin A- or rotenone-treated cells (**antimycin A** and **rotenone** panels, respectively) appear to be intact, forming a well-shaped tubular network. On the contrary, treatment with sorbitol resulted in a punctate morphology of mitochondria, indicative of their distorted function. This effect was once again “revoked” by antimycin A or rotenone, as demonstrated by the intact, almost tubular mitochondrial networks observed in H9c2 cells pre-incubated with these inhibitors and subsequently exposed to sorbitol, in the presence of these compounds (Figure 7: **antimycin A/sorbitol** and **rotenone/sorbitol** vs. **sorbitol** panels). Antimycin A and rotenone on their own had no effect on the mitochondrial pattern (Figure 7: **antimycin A** and **rotenone** panels, respectively).

Cytochrome c release into the extramitochondrial environment is a primary component in the apoptotic cascade and signifies mitochondrial dysfunction [18]. In agreement with our findings thus far, cytochrome c immunoreactivity was only observed in the cytosol of H9c2 cells treated for 1 h with curcumin or sorbitol, while it was obliterated in H9c2 cells exposed to either stress stimulus in the presence of antimycin A or rotenone (Figure S3).

### 2.7. Antimycin A and Rotenone Exert Their Inhibitory Effect on Sorbitol-Induced Apoptosis Independently of ROS Generation

**Figure 8:**
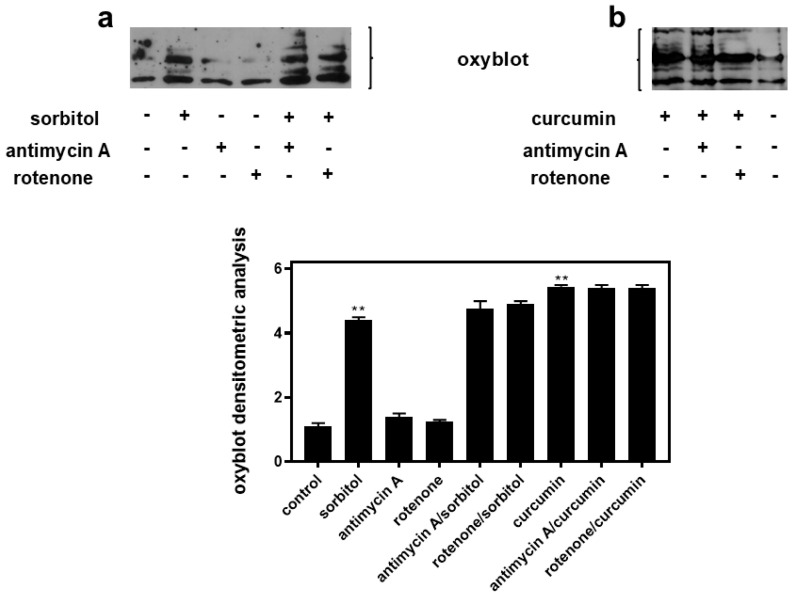
Antimycin A and rotenone do not hinder sorbitol- or curcumin-induced generation of ROS in H9c2 cardiac cells. (**a**,**b**) Oxyblot analysis was performed in H9c2 cells that were left untreated (control), or were exposed to sorbitol (sorbitol), curcumin (curcumin), antimycin A, or rotenone, or to either inhibitor followed by treatment with sorbitol or curcumin, in the presence of the respective inhibitor (antimycin A/sorbitol, antimycin A/curcumin, rotenone/sorbitol, and rotenone/curcumin). After homogenization in RIPA buffer, lysates were incubated with DNPH, proteins were electrophoresed (SDS-PAGE) and immunoblotted with an anti-DNP antibody. Immunoreactive bands were quantified by scanning densitometry and plotted (respective graph). Results are means ± SEM for at least three independent experiments. ** *p* < 0.001 compared to control values.

With the preservation of the mitochondrial morphology and function indicating efficient bioenergetics, we continued by evaluating the potential involvement of ROS levels in the responses observed. The maintenance of mitochondrial function was not due to the obstruction of ROS generation, since an oxyblot analysis revealed that sorbitol- and curcumin-induced ROS levels remained unaltered in the presence of antimycin or rotenone (Figure 8a and b, respectively). Focusing on mitochondrial superoxide levels in particular, we next stained cells with MitoSOX red. As illustrated in Figure S4, the generation of superoxide in the mitochondria of H9c2 cells treated with either sorbitol or curcumin was equally not impeded in the presence of antimycin A nor by rotenone.

### 2.8. Role of Calcium in the Beneficial Effect of Antimycin A Against Sorbitol-Induced Cellular Death in H9c2 Cells

**Figure 9:**
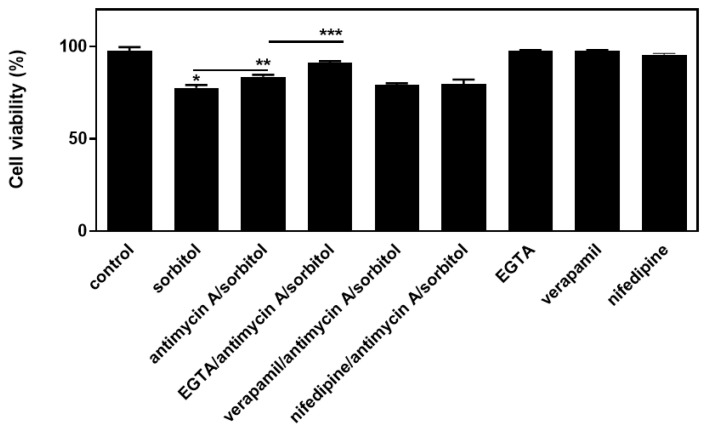
Antimycin A exerts a salutary effect on H9c2 cell viability which appears to be associated with extracellular calcium. Measuring H9c2 (%) cell viability by trypan blue exclusion assay, cells were left untreated (control), treated with sorbitol (sorbitol), or with antimycin A followed by exposure to sorbitol (antimycin A/sorbitol), or exposed to calcium-related compounds (EGTA, verapamil, and nifedipine) alone or before being treated with antimycin A and sorbitol (antimycin A/sorbitol). All experiments were performed in triplicate. Values are means ± SEM for three independent experiments. * *p* < 0.05 compared to control values; ** *p* < 0.01 compared to sorbitol-treated cells; *** *p* < 0.01 compared to cells pre-treated with antimycin A and subsequently exposed to sorbitol in the presence of antimycin A.

These findings advocate for a ROS-independent mechanism, mediating the salutary effect of antimycin A and rotenone. Focusing on the potential involvement of calcium, we observed that chelating extracellular calcium further strengthened cellular viability initially enhanced by antimycin A, in the presence of sorbitol. Hence, as shown in Figure 9, performing the trypan blue exclusion assay, we found that the exposure of H9c2 cells to 0.5M sorbitol for 1 h significantly reduced their viability to 77.5 ± 0.8%. In agreement with the complete inhibition of sorbitol-induced PARP proteolysis by antimycin A observed in Figure 6a, the pre-incubation of H9c2 cells with antimycin A before exposure to sorbitol, augmented cell viability (83.5 ± 0.65%). Evidently, in the presence of EGTA, this beneficial effect of antimycin A was further enhanced (90.5 ± 1.6%). On the other hand, pre-incubation with nifedipine or verapamil did not modulate the beneficial effect of antimycin A against sorbitol exposure. Hence, our results point to a key role of extracellular calcium in modulating cellular viability in our experimental setting, under conditions of hyperosmotic disturbance.

### 2.9. Antimycin A and Rotenone Do Not Interfere with Sorbitol-Induced eIF2α Phosphorylation, Further Contributing to Restoration of Homeostasis

**Figure 10:**
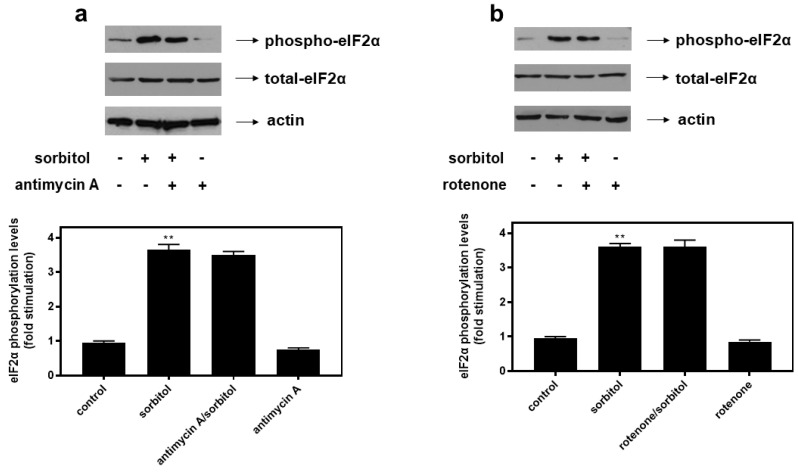
Antimycin A as well as rotenone do not modulate phosphorylation of eIF2α under conditions of hyperosmotic stress in H9c2 cardiac cells. H9c2 cells were left untreated (control), or treated with 0.5 M sorbitol (sorbitol), or with either antimycin A or rotenone alone, or were pre-incubated with either antimycin A or rotenone for 30 min and then exposed to 0.5 M sorbitol in the presence of the inhibitors (antimycin A/sorbitol or rotenone/sorbitol, respectively). (**a**,**b**) Cell extracts (40 μg/lane) were subjected to SDS-PAGE and immunoblotted with antibodies that detect phosphorylated eIF2α (upper panels), total levels of eIF2α (middle panels), or total levels of actin (bottom panels). Western blots are representative of at least three independent experiments with overlapping results. Immunoreactive bands were quantified by laser scanning densitometry and plotted (respective graphs). Results are means ± SEM for at least three independent experiments. ** *p* < 0.01 compared to control values.

Hyperosmotic stress has been shown to trigger the Integrated Stress Response (ISR) evidenced by the phosphorylation of eIF2α at Ser51 [25], attenuating initiation of translation and, hence, promoting restoration of homeostasis [29]. Subsequently, H9c2 cells were left untreated or were incubated with the inhibitors alone, or with the inhibitors followed by exposure to 0.5 M sorbitol for 1 h. Neither antimycin A (Figure 10a upper panel) nor rotenone (Figure 10b upper panel) modulated sorbitol-induced eIF2α phosphorylation levels, indicating preservation of this cellular defensive strategy against the hyperosmotic insult. Total eIF2α protein levels were found to be constitutively expressed (Figure 10a,b: middle panels). Equal protein loading was verified by the immunodetection of actin levels (Figure 10a,b: bottom panels).

## 3. Discussion

Mitochondria hold a central role as hubs of energy metabolism in eukaryotic cells. Nevertheless, they are not only involved in ATP generation but also regulate a number of biological processes, including cell-death-associated mechanisms [16]. Therefore, in the present study, we decided to probe into the potential role of mitochondria in the deleterious effect conferred by curcumin [23] and sorbitol [24,25], previously demonstrated in H9c2 cardiac cells, given the significance that the maintenance of cardiac cells’ homeostasis has under stress conditions, promoting preservation of myocardial function.

To this end, we initially confirmed the early onset of the apoptotic mechanism in H9c2 cells after 1 h of exposure to 50 μM curcumin, by the detection of PARP proteolysis, which routinely serves as an apoptotic marker (Figure 1a). Cleavage of PARP between Asp214 and Gly215 blocks recruitment of its catalytic domain to sites of DNA damage [17]. In addition, observing Bax translocation from the cytosol to the mitochondria (Figure 1d–g) underlined the initiation of the intrinsic apoptotic pathway. In agreement with our results, Shu et al. have reported curcumin to trigger apoptosis in hepatic stellate cells [30], with many other studies also noting curcumin-induced apoptosis mainly in cancer cells [31,32]. Aiming to identify effectors able to counteract this detrimental impact of curcumin on H9c2 viability, a Western blot analysis revealed that MAPK inhibitοrs as well as established antioxidants failed to do so (Figure S1). This finding contradicts studies noting the suppression of apoptosis in the presence of JNK inhibitors in H9c2 cells [23], HT-29 colon carcinoma cells [33], and THP-1 human monocytic leukemia cells [34]. In addition, NAC has been found to inhibit apoptosis in A549 lung adenocarcinoma cells [35], and HCT116 human colon cancer cells [36], as well as in keratinocytes [37]. These discrepancies could be cell-type-specific or attributed to the concentration of curcumin used in our study [50 μM], possibly triggering different signaling effectors and routes.

Further monitoring the possibility of ROS involvement in the observed responses, HOE642, a specific inhibitor of NHE-1, as well as apocynin, an inhibitor of Nox were equally found not to modulate curcumin-induced PARP cleavage as shown in Figure 2a,b. This finding is not consistent with the established notion regarding the cardioprotective effects of NHE-1 [38] as well as NOX inhibition [39]. Nevertheless, in line with our observations, studies also exist which underline apocynin’s potential cytotoxic impact [40], along with the lack of a salutary effect of NHE-1 inhibition in the event it coincides with a functional Na^+^/K^+^ ATPase (preventing ionic homeostasis dysregulation) [38].

Keeping in mind that the mitochondrial respiratory chain also constitutes a major source of ROS in cardiac myocytes [41], the effect of antimycin A, an inhibitor of electron transport chain complex III, was assessed. Surprisingly, in the presence of 0.1 μM antimycin, curcumin-induced PARP proteolysis was totally shut off (Figure 2c,d). Concomitantly, 0.1 μM of antimycin A reversed curcumin-stimulated DNA condensation (Figure S2) and also enhanced H9c2 cells’ viability under the conditions investigated (Figure 2e). The salutary effect of antimycin A was also evident using Mitotracker, so as to assess the mitochondrial structural integrity (Figure 3). Thus, the fragmented and punctate pattern of mitochondria caused by curcumin was restored in the presence of antimycin A, indicating mitochondrial recovery. Along this line, antimycin A was also found to offer significant protection against econazole-induced cell death, in human promyelocytic leukemia HL-60 cells [42] via the suppression of apoptosis. Econazole is an ER stress agent that suppresses calcium influx and stimulates ER calcium depletion, while reinforcing oxidative stress in cells. Likewise, antimycin inhibited nitric-oxide-induced apoptosis in a rat gastric epithelium cell line, with NO’s cytotoxicity closely linked to mitochondrial dysfunction [43]. Similarly, the transient inhibition of mitochondrial ETC complex III by another inhibitor, myxothiazol, in the human RKO carcinoma cell line also promoted cell survival by attenuating apoptosis [44]. On the contrary, in potassium-iodide-treated FRTL cells, antimycin A was observed to stimulate rather than attenuate apoptosis, leading to the upregulation of oxidative stress and mitochondrial dysfunction [45]. These discrepancies regarding antimycin’s impact could be cell-type- or stimulus-specific, or ascribed to different concentrations of the compound used, setting off diverse responses.

Mitochondrial metabolism and activity are closely interconnected with calcium signaling [46]. Calcium uptake through the mitochondrial calcium uniporter (MCU), as well as calcium efflux via the Na^+^–Ca^2+^–Li^+^ exchanger, in conjunction with the sodium–proton exchanger (NHE), along with calcium buffering by calcium phosphate, determine calcium equilibrium in mitochondria [47]. Taking under consideration the fact that mitochondrial calcium levels finetune the bioenergetic metabolism with cellular requirements, but may also promote cell death [48], we next assessed the effect of calcium regulators on the protection conferred by antimycin A (Figure 4). Chelation of extracellular calcium by EGTA further enhanced the protection afforded by antimycin, indicating that, when calcium originating from the external milieu is unavailable to enter the cell (via receptor-mediated pathways), cellular viability is enhanced. Indeed, cell death has been associated with calcium overload, as well as perturbations of intracellular calcium compartmentalization [49]. Hence, necrotic cell death has been attributed to intracellular Ca^2+^ overload, while apoptotic as well as other forms of cell death mechanisms, are associated with an excessive calcium mitochondrial influx originating from the cytoplasm [50].

On the other hand, blockade of the voltage-dependent L-type calcium channels (LTCCs) by verapamil or nifedipine cancelled antimycin’s protective effect on curcumin-treated H9c2 cells (Figure 4). This finding underlines a potential interconnection between these high-voltage activated calcium channels and mitochondrial ETC function. Hence, one could postulate that the repression of curcumin-induced cell death due to the blockade of mitochondrial complex III with antimycin A is related to LTCC activation. In agreement with our data, LTCCs have been ascribed as “redox sensors”, with their activity being controlled by mitochondria [51]. In addition, our intriguing finding highlights the key role that functional LTCCs play in the preservation of calcium equilibrium under the experimental conditions tested, with their blockade exerting a cytotoxic effect. The latter could be interrelated with the perturbation of the balance accomplished by LTCCs and all the other effectors (channels, pumps, and macromolecular complexes) involved in calcium homeostasis. One should also keep in mind that the different populations of LTCCs that exist, feature distinct properties and diverse modes of affecting the calcium flux, ranging from modulating the sarcoplasmic reticulum calcium loading, to activating the calcium-induced calcium release mechanism. Another module is LTCCs’ sub-cellular localization in T-tubule dyadic complexes with calcium-release channels, that are directly associated with the mitochondrial outer membrane (reviewed in [52]).

Next, curcumin-stimulated PARP fragmentation was found to also be abolished in the presence of 0.1 μM rotenone (Figure 5). Supporting our findings, the beneficial effect of rotenone has also been reported in diabetic mice where it alleviated hyperglycemia [9], in mice where it limited lipopolysaccharide/D-galactosamine-induced liver injury [10], and in aldosterone-infused rats where it repressed renal injury [11], as well as in mice where it attenuated deoxycorticosterone acetate-salt induced hypertension [53]. Recent evidence also accounts for the beneficial impact of rotenone against β-cell apoptosis in a streptozotocin (STZ)-induced model of Type 1 diabetes mellitus as well as in Min6, a cultured mouse pancreatic β-cell line [54]. One should, however, also report upon studies that have demonstrated rotenone to exert a degrading effect, further enhancing apoptosis in FRTL cells exposed to high concentrations of iodide [45]. Additionally, it was also observed to upregulate dichloroacetate cytotoxicity in VM-M3 glioblastoma cells [55]. These discrepancies may be cell-type- or concentration-dependent, attributed to the diverse routes of administration applied, or could, once again, signify triggering of different signal transduction pathways, consistent with the complex physiology and multilevel interplay of mitochondria with other subcellular compartments and organelles [56]. At this point, one should account for the fact that, although curcumin-induced PARP proteolysis in the presence of 0.1 μM rotenone is practically obliterated (Figure 5a,b), cell viability is significantly enhanced, but not to a respectively extensive degree (Figure 5c). This discrepancy could be due either to the consistent ROS levels that remain unchanged under the conditions studied (Figure 8b and Figure S4), or to other signaling pathways possibly activated in parallel, hindering a full reversal of the deleterious effect of curcumin. One should also consider that the suppression of apoptosis has often been shown to promote the transition to other pro-death mechanisms, i.e., necroptosis [57], pyroptosis [58] etc.; further studies are required to fully elucidate the mechanisms mediating these responses.

Subsequently, we probed into the respective effects of antimycin A and rotenone on apoptosis, stimulated under conditions of hyperosmotic stress. Inhibiting mitochondrial complex III as well as complex I equally obliterated PARP fragmentation in H9c2 cells treated with 0.5 M sorbitol (Figure 6). Consistently, sorbitol-induced mitochondrial fragmentation, a morphological hallmark of apoptosis, was reversed in H9c2 cells, in the presence of either of the inhibitors, with filamentous mitochondria “re-occupying” the cytoplasm of antimycin/sorbitol- as well as rotenone/sorbitol-treated H9c2 cells (Figure 7). Of interest, this finding is compatible with the restoration of mitochondrial function, evidenced by the abolition of sorbitol- as well as curcumin-induced cytochrome c release into the cytosol of H9c2 cells, in the presence of antimycin A or rotenone (Figure S3). The cytochrome c release into a cell’s cytoplasm is established to coincide with a significant drop in Δψ (MMP collapse), the permeabilization of the outer mitochondrial membrane, and a loss of ATP-producing capacity and mitochondrial integrity [59]. *Could this recovery be attributed to the limited number of intact mitochondria that managed to resist the stress insult*? Tait et al. have highlighted the fundamental contribution of this “critical pool” to re-construct the cellular mitochondrial network, promoting survival in response to various stimuli and in different cell types [60]. *Could the recovery noted be attributed to “anastasis”*? The dampening of the apoptotic cascade, termed “anastasis” from the Greek word meaning “resurrection”, has been observed in primary heart, brain, and liver cells, macrophages, NIH 3T3 fibroblasts, and HeLa cells in a study by Tang et al. However, this cell resuscitation took place after the withdrawal of the apoptotic insult (ethanol was washed away), leaving an oncogenic fingerprint [61]. In this study, researchers postulate that, specifically for terminally differentiated cells that cannot be replaced after injury, anastasis promotes their preservation, on the grounds that conditions improve. In addition, Narula et al. have observed heart cells to maintain their nuclear integrity, halting and reversing the apoptotic signaling cascade in the context of heart failure, “seeing hope in death”, as they characteristically underline [62]. Furthermore, in correlation with our data, a reversal of the intrinsic apoptotic pathway triggered by doxycycline was observed in the human osteosarcoma-U2OS-derived cell line, along with the recovery and maintenance of mitochondria crista structure, in the presence of a compound that was verified to target the succinate dehydrogenase subunit B (SDHB) protein, a component of complex II of the mitochondrial ETC [63]. With mitochondrial crista consisting of folds of the inner mitochondrial membrane that accommodate the ETC components, the activity of the chain is directly linked to the dynamic remodeling of the crista.

In addition, our oxyblot analysis and staining cells with MitoSOX red revealed that the enhancement in survival in the presence of antimycin A and rotenone was not associated with the repression of ROS generation levels since the latter was not altered in the presence of the inhibitors (Figure 8 and Figure S4). This result agrees with the established notion that, rather than repressing ROS generation, the inhibition of the mitochondrial ETC by antimycin A or rotenone may result in augmenting ROS levels [6]. The stable ROS levels observed in our study could be attributed to the low concentration at which the compounds are used, also justifying the absence of any cytotoxicity upon H9c2 cells. On the other hand, the crucial role of calcium influx was once again confirmed, since the beneficial effect of antimycin A was enhanced in sorbitol-exposed H9c2 cells pre-treated with EGTA, while LTCCs were not found to participate in the observed responses (Figure 9). To further support our data, Kon et al. have pointed out the beneficial role of DS44170716, a small-molecule compound, that was found to protect human liver HepG2 cells against ionophore A23187-triggered death. This effect was conferred via hindering calcium intake and overload, in conjunction with strongly inhibiting mitochondrial complex III and weakly inhibiting complex IV and V [64].

**Figure 11:**
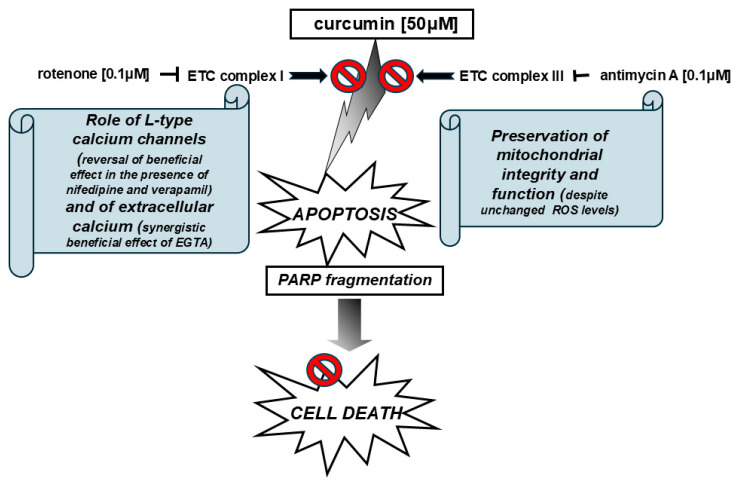
Schematic diagram illustrating inhibition of curcumin-induced apoptosis and cell death in H9c2 cells by rotenone and antimycin A, via preservation of mitochondrial integrity and function through signal transduction mediators including extracellular calcium and L-type calcium channels. 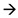 activation; 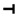 inhibition. 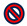 inhibition.

**Figure 12:**
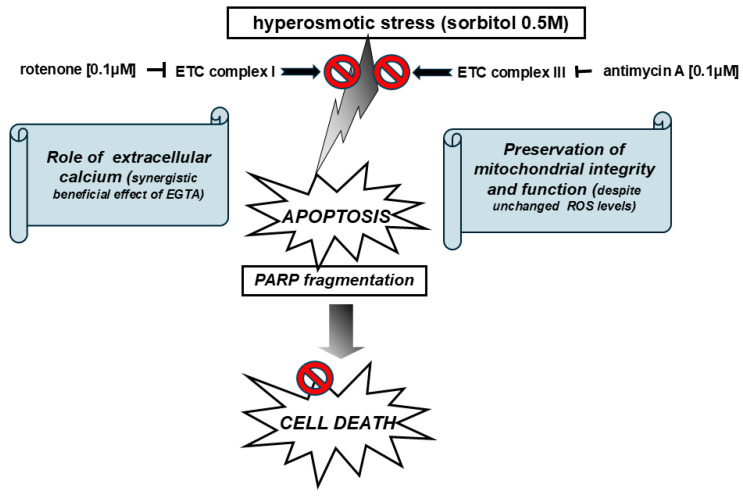
Schematic diagram illustrating inhibition of sorbitol-induced apoptosis and cell death in H9c2 cells by rotenone and antimycin A, via preservation of mitochondrial integrity and function through signal transduction mediators including extracellular calcium. 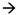 activation; 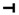 inhibition. 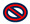 inhibition.

Overall, our findings argue for hyperosmotic-stress-induced PARP proteolysis and the ensuing features of the apoptotic pathway to come to a complete halt and to be reversed in the presence of antimycin A as well as rotenone. Thus, it was interesting to also determine their impact on an adaptive pro-survival pathway, triggered under these conditions [25], the Integrated Stress Response (ISR). In this context, antimycin A as well as rotenone failed to confer any alteration on sorbitol-stimulated eIF2 alpha phosphorylation levels (Figure 10), a characteristic marker of the IRS which leads to a decline in de novo protein synthesis [26]. This finding further supports the notion that these two mitochondrial respiratory chain inhibitors hold a beneficial role in our experimental model, preserving the activation of a compensatory signaling pathway and, at the same time, blocking a cell death modality. The schemes illustrated in Figure 11 and Figure 12 outline the main effectors which mediate the salutary effect of blocking mitochondrial complex III (by antimycin A) and mitochondrial complex I (by rotenone) on H9c2 cells’ viability, promoting their survival to a partial but significant degree.

Supplemental studies are, nevertheless, required to delineate, in depth, the mode of action of these compounds, given the complex and dynamic nature of mitochondrial physiology and function. In this context, given that mitochondrial quality control through mitophagy consists of another fundamental mechanism, ensuring mitochondrial homeostasis, the determination of its potential contribution to the observed responses in future studies could also be worthwhile. At this point, one must admit that extrapolating our findings to the context of cardiac pathophysiological conditions is highly speculative, bearing in mind the constant changes in the intracellular as well as the mitochondrial milieux, along with the fluctuating concentrations of metabolites and activities of enzymatic moieties involved. Hence, the contribution of antimycin A and rotenone to the preservation of cell integrity and homeostasis remains to be determined. Nevertheless, our study clearly indicates that these compounds, which have already been extensively studied and used in multiple applications, could be exploited as agents conferring cardioprotection against severe stress insults, through preservation of mitochondrial function and calcium overload blockade. Yet again, one should keep in mind the limitations that may apply, given that tissue culture cells and cardiac cells possess different alternative metabolic pathways they may switch to, so as to accommodate their energetic demands under stressful conditions.

## 4. Materials and Methods

### 4.1. Reagent and Antibodies

Dithiothreitol (DTT), phenylmethylsulphonyl fluoride (PMSF), dimethyl sulfoxide (DMSO), D-sorbitol, and Bradford protein assay reagent were purchased from AppliChem GmbH (Darmstadt, Germany). Leupeptin, trans-epoxysuccinyl-L-leucylamido-(4-guanidino) butane (E-64), Thiazolyl Blue Tetrazolium Bromide (MTT), nifedipine, verapamil, and trypan blue (0.4% *w*/*v*) were from Merck (Merck KGaA, Darmstadt, Germany). Curcumin was purchased from Cayman Chemical (AnnArbor, MI, USA). EGTA, HOE642, apocynin, *N*-Acetyl-L-cysteine, SOD, antimycin A, and rotenone were purchased from Sigma-Aldrich (St. Louis, MO, USA).

Nitrocellulose (0.45 μm) was obtained from Macherey-Nagel GmbH (Duren, Germany). Prestained molecular mass markers were from New England Biolabs (Beverly, MA, USA). The rabbit antibodies for poly (ADP-ribose) polymerase (PARP) (#9542), eIF2α (#9722), Bax (#2772), phospho-eIF2α (Ser51) (#9721), and GAPDH (#2118) were purchased from Cell Signaling Technology Inc. (Beverly, MA, USA), while rabbit polyclonal anti-actin (A2103) was from Merck (Burlington, MA, USA). TOMM20 polyclonal antibody (#11802-1-AP) was purchased from Proteintech (Manchester, UK). SB203580, AS600125, and U0126 were also from Merck. The peroxidase-conjugated goat anti-rabbit IgG secondary antibody (#AP132P) was from Merck. The enhanced chemiluminescence (ECL) kit was from GE Healthcare (Buckinhamshire, UK). Super RX film was purchased from Fuji photo film GmbH (Dusseldorf, Germany). Cell culture supplies were from PAA Laboratories (Pasching, Austria). Oxyblot Protein Oxidation Detection Kit was from Merck. Hoechst 33258, Mitotracker RED CMXRos, and MitoSOX Red were purchased from Invitrogen, Thermo Fisher Scientific (Waltham, MA, USA).

### 4.2. Cell Culture and Treatments

H9c2 rat cardiac myoblasts (passage 18–25; American Type Culture Collection CRL-1446, Manassas, VA, USA) were grown in medium containing high-glucose (4.5 g/L) Dulbecco’s modified Eagle’s medium (DMEM; Gibco, Paisley, UK) in the presence of 10% (*v*/*v*) fetal bovine serum (FBS; ThermoFischer Scientific, Waltham, MA, USA) and penicillin-streptomycin (ThermoFischer Scientific, Waltham, MA, USA), under a humidified atmosphere of 95% air/5% CO_2_ at 37 °C. Cells were seeded in 60 mm dishes and grown to approximately 70–80% confluence. Serum had been withdrawn for at least 18 h before performing any treatment. Cells were treated with curcumin (50 μM) or sorbitol (0.5 M) for the times indicated. All inhibitors used were dissolved in DMSO and added to the medium 30 min prior to treatment with curcumin or sorbitol. Hence, H9c2 cells were left untreated (control), incubated with the inhibitors alone, or with the inhibitors followed by exposure to curcumin or sorbitol, for the times indicated.

### 4.3. Cell Viability Assays

The number of viable cells was determined using the trypan blue exclusion assay. Trypan Blue Staining Solution traverses the membrane of non-viable cells, coloring them blue. Therefore, after seeding H9c2 cells in 60 mm dishes, cells were left untreated (control), treated with the inhibitors alone or with the inhibitors followed by exposure to curcumin (50 μM) or sorbitol (0.5 M). All experiments were performed at least in triplicate. After treatments, cells were rinsed with PBS and harvested with trypsin. So as to evaluate the percentage of viable cells, the cell suspension was subsequently mixed with 0.4% (*w*/*v*) trypan blue solution (in a 5:1 ratio) and further incubated for 2 min, before being loaded onto a hemacytometer and counted under a light microscope. Percentage of viable cells was calculated as follows:

% viable cells = (number of unstained cells/total number of cells) × 100

### 4.4. Protein Extraction

After completion of treatments, cells were washed with ice-cold phosphate-buffered saline (PBS) and harvested. For whole-cell extracts, H9c2 cells were lysed in ice-cold buffer containing the following: 20 mM Tris-HCl pH 7.5, 20 mM β-glycerophosphate, 2 mM EDTA, 10 mM benzamidine, 20 mM NaF, 0.2 mM Na_3_VO_4_, 200 μM leupeptin, 10 μM E-64, 5 mM DTT, 300 μM PMSF, and 0.5% (*v*/*v*) Triton X-100 and left on ice for 15 min. After lysates were centrifuged (BR4i Jouan centrifuge, 20,800× *g*, 10 min, 4 °C), the supernatants (total protein extract) were collected. Protein concentration was determined using the Bradford assay. After quantification, 0.33 vol. of sodium dodecyl sulphate (SDS) sample buffer [SB4X: 0.33 mol/L Tris-HCl (pH 6.8), 10% (*w*/*v*) SDS, 13% (*v*/*v*) glycerol, 20% (*v*/*v*) 2-mercaptoethanol, 0.2% (*w*/*v*) bromophenol blue] was added to the samples, which were subsequently boiled and stored until use at −20 °C. Levels of Bax were analyzed in mitochondrial and cytosolic cell fractions. Mitochondria were isolated according to the method described by Gottlieb and Granville (2002) [65], with slight modifications. In particular, cells were extracted in buffer M [250 mM sucrose, 20 mM Hepes, 10 mM KCl, 1.5 mM MgCl_2_, 0.1 mM EDTA, 1 mM EGTA, 1 mM DTT, and 200 μM PMSF] and incubated at 4 °C for 20 min. Extracts were centrifuged twice (5000× *g*, 5 min, 4 °C) and the resulting supernatants were centrifuged at 10,000× *g* for 15 min (4 °C). Next, pellets were re-suspended in buffer M containing 0.1% (*v*/*v*) Triton X-100, and centrifuged (20,800× *g*, 15 min, 4 °C), and protein concentrations were determined using the Bradford assay. In addition, samples were boiled with 0.33 vol. of SB4X. For evaluation of caspase-3 activation, cells were homogenized with Chaps buffer [50 mM HEPES/KOH pH 6.5, 2 mM EDTA, 0.1% (*w*/*v*) Chaps, 20 lg/mL leupeptin, 5 mM DDT, 1 mM PMSF, 10 lg/mL aprotinin, and 10 lg/mL pepstatin A]. Samples were subsequently repeatedly frozen [−80 °C (× 3)] and left to thaw. Lysates were next centrifuged (20,800× *g*, 4 °C, 20 min) and protein concentrations were once again determined using the Bradford assay. Samples were then boiled with 0.33 vol. of SB4X.

### 4.5. Oxyblot Protein Oxidation Detection

Protein oxidation was detected by reaction with 2,4-dinitrophenyl hydrazine (DNP) using an OxyBlot™ Protein Oxidation Detection Kit (Merck, KGaA, Darmstadt, Germany). Briefly, after seeding H9c2 cells in 60 mm dishes, cells were left untreated (control), treated with antimycin A or rotenone alone, or with antimycin A or rotenone, followed by exposure to curcumin (50 μM) or sorbitol (0.5 M), or treated with curcumin (50 μM) or sorbitol (0.5 M) for 1 h. All experiments were performed at least in triplicate. After treatments, cells were rinsed with PBS, homogenized in RIPA buffer [50 mM Tris-HCl pH 7.5, 150 mM NaCl, 1% (*v*/*v*) Triton X-100, 0.1% SDS], and incubated on ice for 15 min. Lysates were centrifuged in a BR4i Jouan centrifuge (14,000× *g*, 5 min, 4 °C) and the supernatants were collected. Protein concentration was determined using the Bradford assay. Carbonyl groups in the protein side chains were derivatized to DNP-hydrazone by reaction with DNPH. Proteins were next electrophoresed on an SDS-PAGE gel, followed by immunoblotting with an anti-DNP antibody (1:150) so as to detect carbonyl groups.

### 4.6. Detection of Mitochondrial Superoxide Levels

MitoSOX compounds constitute novel, live-cell-permeant, fluorogenic dyes specifically targeted to mitochondria. When MitoSOX Red indicator is oxidized by mitochondrial superoxide, red fluorescence is produced. Thus, so as to determine the levels of mitochondrial superoxide by fluorescence microscopy, H9c2 cells were grown in ibidi µ-Slide 4 Well coverslips. Before all treatments, serum was withdrawn for at least 18 h. Next, H9c2 cells were left intreated (control), or treated with sorbitol (0.5 M), or antimycin A (0.1 μM), or were pre-incubated with antimycin A for 30 min and subsequently exposed to sorbitol for 1 h, in the presence of the inhibitor (antimycin/sorbitol). Cells were then incubated with MitoSOX Red (5 µM in serum-free DMEM) for 10 min at 37 °C. After washing twice in PBS, DMEM was added in the wells and cells were visualized under a Zeiss Axioplan microscope. Digitized images were acquired with a Zeiss Axiocam MRc5 digital camera with the AxiovisionRel 4.4 image software (Edition 2005). MitoSOX intensity was evaluated using ImageJ software version 1.54g (NIH, Bethesda, MD, USA).

### 4.7. Chromatin Condensation Detection Assay

H9c2 cells were cultured onto coverslips in 24-well plate chambers and treated with 50 μM of curcumin for 1 h. Untreated cells served as a control group. After treatment, medium was removed and 10 μg/mL Hoechst 33258 was added to each well (15 min incubation). Coverslips were then washed three times with PBS, placed on glass slides, and covered with mounting medium (P-PDA: p-phenylen-diamine). Apoptosis was evident in cells with characteristic bright, condensed nuclei. Visualization of nuclear morphology and fluorescence was performed using a Zeiss Axioplan microscope (Carl Zeiss Microscopy, LLC, One North Broadway, NY, USA). Digitized images were acquired with a Zeiss Axiocam MRc5 digital camera with the AxiovisionRel 4.4 image software (Edition 2005).

### 4.8. Fluorescence Viewing of Mitochondria with MitoTracker Red CMXRos

MitoTracker probes are cell-permeant mitochondrial stains that contain a mildly thiol-reactive chloromethyl moiety, suitable for labeling mitochondria. In particular, after incubation, MitoTracker dye passively diffuses across the plasma membrane and is accumulated in active mitochondria of live cells. H9c2 cells were once again grown in ibidi µ-Slide 4 Well coverslips. Before all treatments, serum was withdrawn for at least 18 h. Subsequently, H9c2 cells were left untreated (control), or treated with sorbitol (0.5 M), curcumin (50 μM), antimycin A (0.1 μM), or rotenone (0.1 μM), or were pre-incubated with the mitochondrial chain inhibitors, followed by exposure to curcumin or sorbitol in the presence of the inhibitors. After washing three times with PBS, H9c2 cells were incubated with Mitotracker red CMXRos (100 nm) for 30 min in the dark (Rt). Cells were washed with PBS three times and were subsequently imaged in PBS using a Zeiss Axioplan fluorescence microscope (Carl Zeiss Microscopy, LLC, One North Broadway, NY, USA).

### 4.9. SDS-PAGE and Immunoblot Analysis

Protein samples containing equal amounts of protein (40 μg) were resolved by SDS-PAGE on 8% (*w*/*v*), 10% (*w*/*v*), or 12% (*w*/*v*) polyacrylamide gels and transferred onto nitrocellulose membranes (0.45 μm). After blocking in Tris-buffered saline Tween (TBST) containing 5% (*w*/*v*) non-fat milk powder (60 min, room temperature), membranes were incubated overnight with the appropriate antibody, according to the manufacturer’s instructions (at 1:1000 dilution). After incubating blots with the respective horseradish peroxidase-conjugated secondary antibody (1:5000 dilution), they were developed with enhanced chemiluminescence (ECL) using the Luminata™ Crescendo Western HRP Substrate (Millipore, Billerica, MA, USA) and quantified by scanning densitometry (Gel Analyzer v. 1.0). Equal protein loading was verified by probing membranes with an anti-actin antibody. Normalization was carried out by dividing the average value of each protein studied, with the respective levels of the control protein in each sample.

### 4.10. Statistical Evaluations

Western blots shown are representative of at least three independent experiments. Data shown correspond to the mean ± SEM and were analyzed by one-way ANOVA multiple-comparison test (Graph Pad Prism Software, San Diego, CA, USA) with group comparisons performed using the Bonferroni post hoc test. *p* < 0.05 was considered to indicate a statistically significant difference.

## Data Availability

The data presented in this study are available upon request from the corresponding author.

## Supplementary Materials

The following supporting information can be downloaded at: https://www.mdpi.com/article/10.3390/ijms26062435/s1.

## Abbreviations

BSA	bovine serum albumin
DMSO	dimethyl sulphoxide
DTT	ditheiothreitol
ECL	enhanced chemiluminescence
CVDs	cardiovascular disorders
ISR	Integrated Stress Response
MAPK	mitogen-activated protein kinase
PARP	poly(ADP-ribose) polymerase
ROS	reactive oxygen species
TBS	Tris-buffered saline
ETC	electron transport chain
EGTA	ethylene glycol-bis(β-aminoethyl ether)-N,N,N′,N′-tetraacetic acid
