# Supplementary material for: “Villains” Turning Good: Antimycin A and Rotenone, Mitochondrial Respiratory Chain Inhibitors, Protect H9c2 Cardiac Cells Against Insults Triggering the Intrinsic Apoptotic Pathway
Source: Int J Mol Sci. 2025 Mar 8;26(6):2435. doi: 10.3390/ijms26062435 (PMC11942121; doi:10.3390/ijms26062435)
Supplement: Supplementary file 1 [file ijms-26-02435-s001.zip › ijms-3487021-supplementary.pdf]

Figure S1 **Zikaki et al. (2025)**

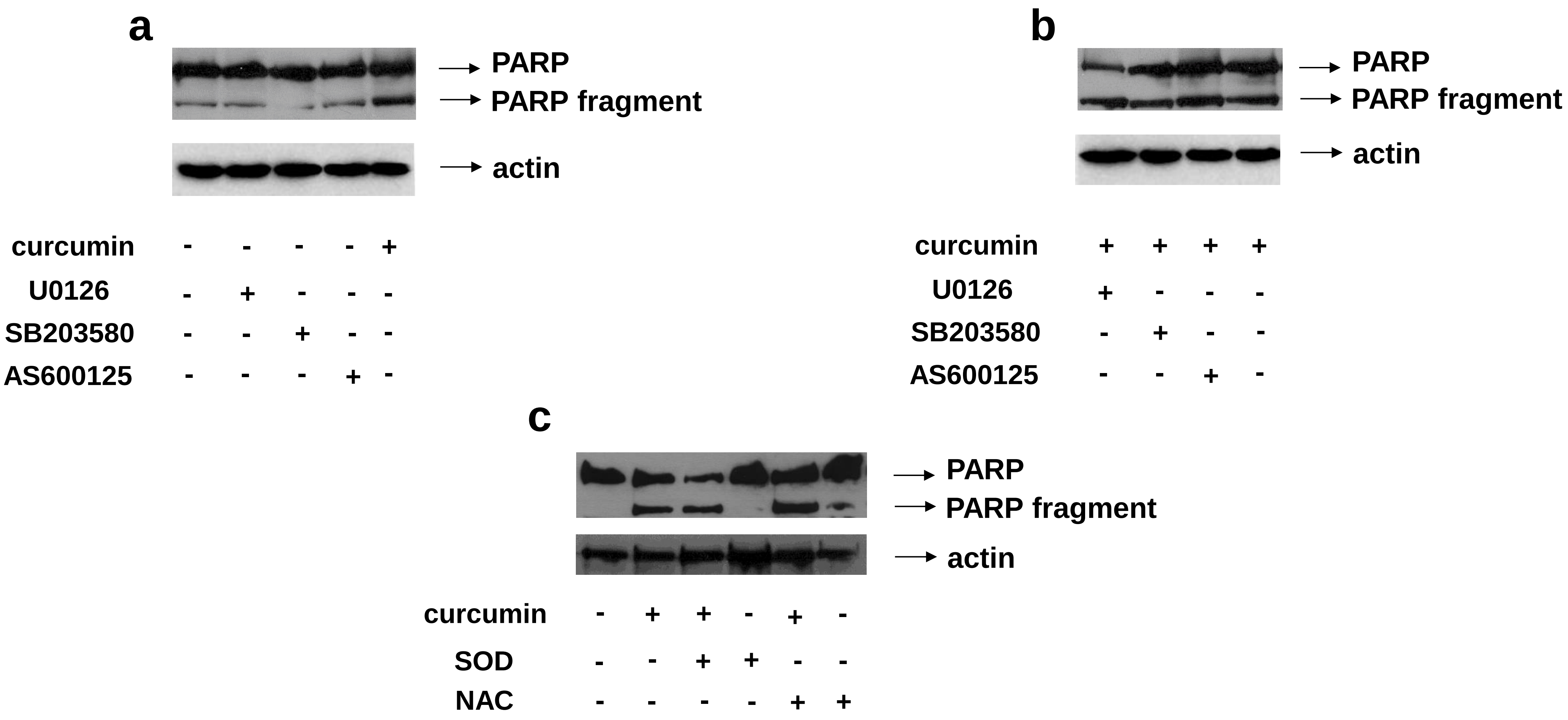

Figure S1. MAPKs inhibitors as well as antioxidants cannot suppress curcumin-induced PARP fragmentation in H9c2 cardiac cells. H9c2 cells were left untreated, or treated with 50μM curcumin, or incubated with U0126, SB203580, AS600125, SOD and NAD alone, or were pre-incubated with these compounds for 30 min followed by exposure to 50 μM curcumin in the presence of the inhibitors, respectively. Cell extracts (40 μg/lane) were subjected to SDS-PAGE and immunoblotted with antibodies that detect PARP (full length and fragment, upper panels), or total levels of actin (bottom panels). Western blots are representative of at least three independent experiments with overlapping results.

Figure S2 **Zikaki et al. (2025)**

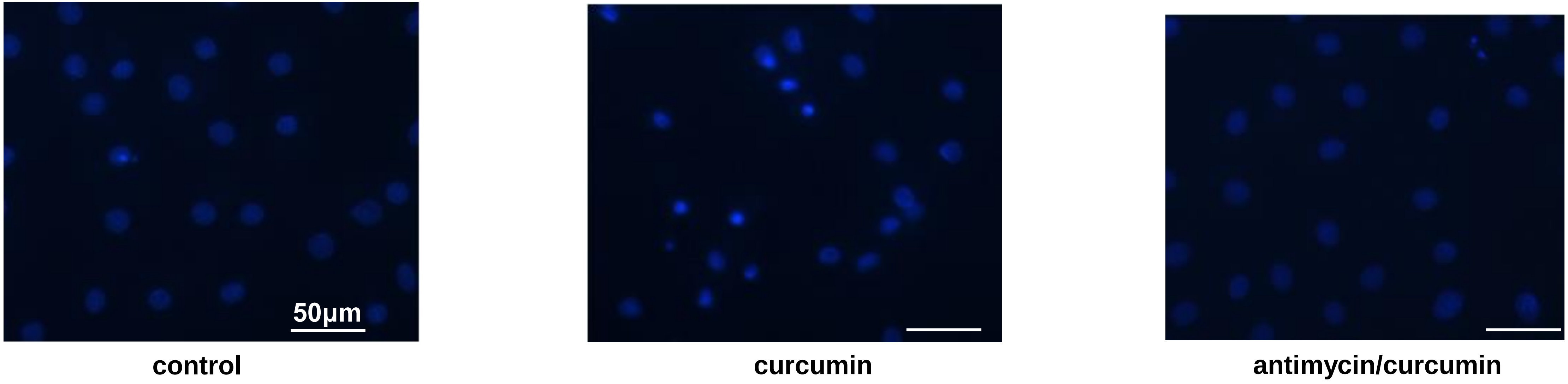

Figure S2. Curcumin stimulates DNA condensation in H9c2 cells counteracted by antimycin A. H9c2 cells were seeded on ibidi chambered μ-slides and incubated in serum-free culture medium. They were left untreated (control), or were incubated with 50μM curcumin, or incubated with antimycin A (0.1μM) followed by exposure to 50μM curcumin, in the presence of the inhibitor (antimycin/curcumin). After treatment, medium was removed, and 10 μg/mL Hoechst 33258 was added to each well (15 min incubation). Coverslips were then washed three times with PBS and covered with mounting medium. Nuclear fluorescence was visualized using a Zeiss Axioplan microscope. Representative photographs for each condition under investigation are illustrated, representative from at least three independent experiments (scale bar 50μM).

Figure S3 Zikaki et al. (2025)

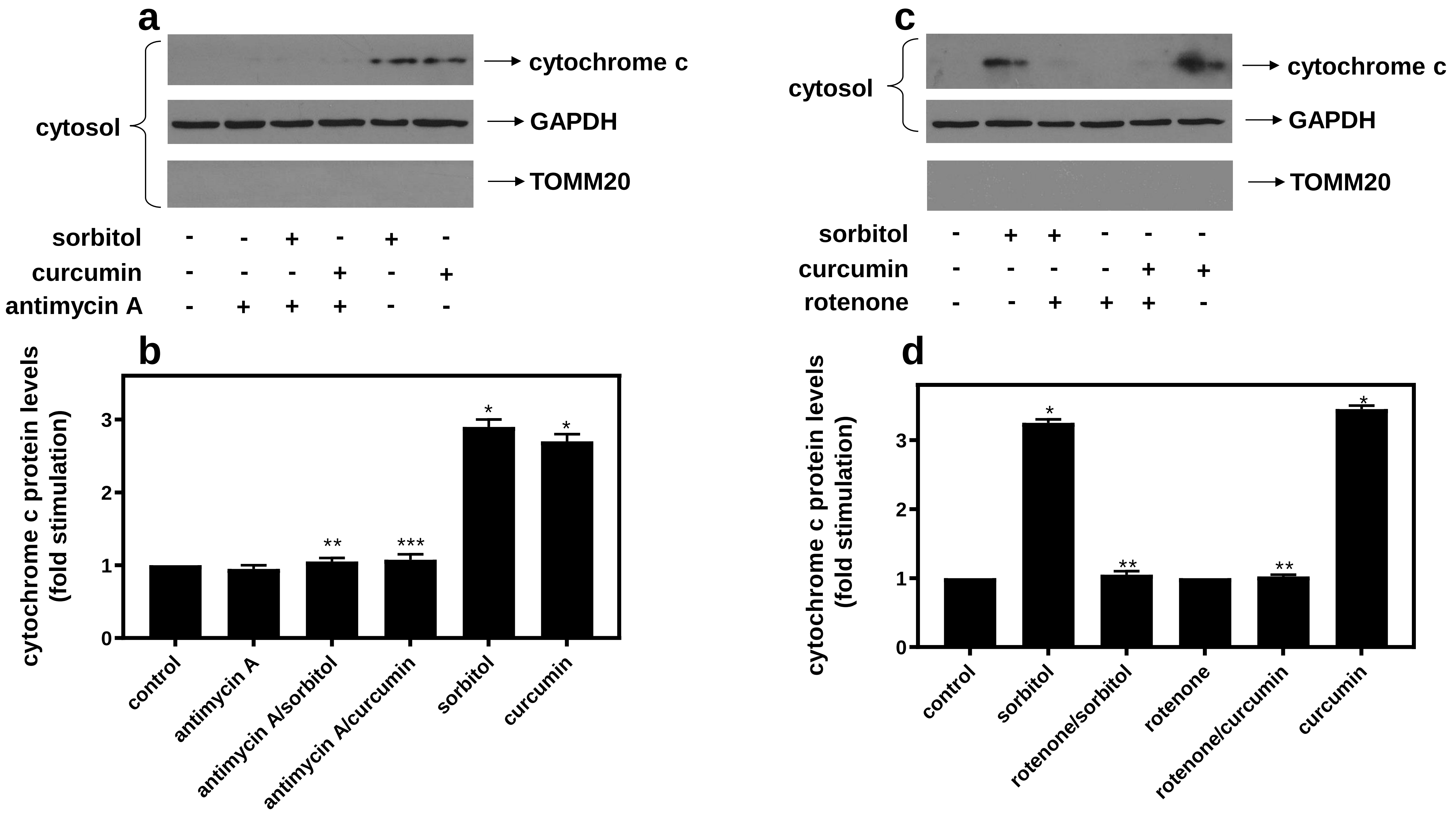

Figure S3. Antimycin A (a, b) as well as rotenone (c, d) both equally eliminate sorbitol– and curcumin-induced cytochrome c release into the cytosol of H9c2 cells. H9c2 cells were left untreated or were incubated with antimycin A or rotenone alone, or with the inhibitors followed by exposure to sorbitol or curcumin in the presence of the inhibitors. Cell extracts (30 μg/lane) were subjected to SDS-PAGE and immunoblotted with antibodies that detect cytochrome c (upper panels), GAPDH (middle panels) as well as TOMM20 (bottom panels). Western blots are representative of at least three independent experiments with overlapping results. Immunoreactive bands were quantified by scanning densitometry and plotted (b and d, respectively). Results are means ± SEM for at least three independent experiments. *$p<0.01$ compared to control values; **$p<0.01$ compared to sorbitol-treated cells in the absence of the inhibitors; *** $p<0.01$ compared to curcumin-treated cells in the absence of the inhibitors.

Figure S4 Zikaki et al. (2025)

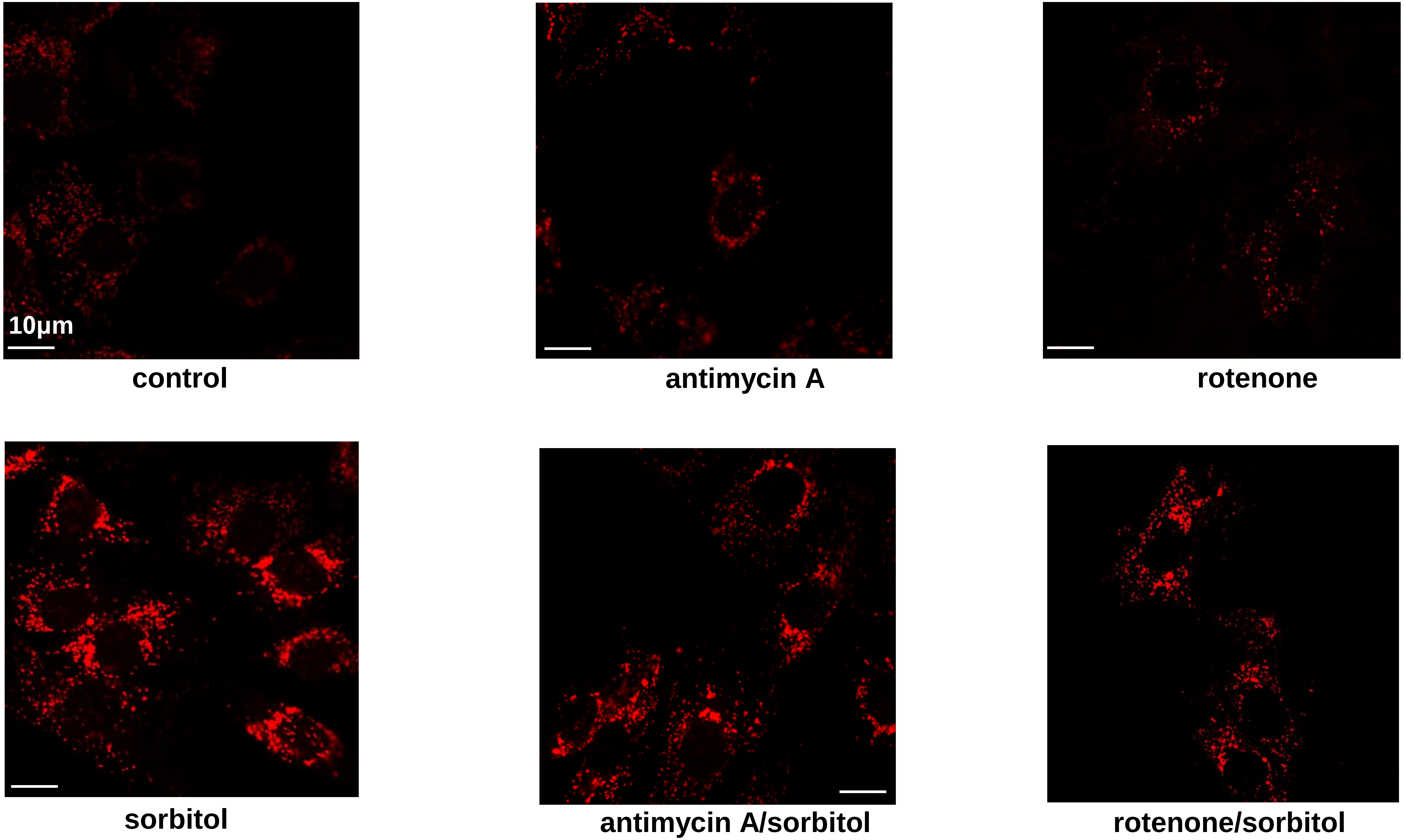

Figure S4 (continued) Zikaki et al. (2025)

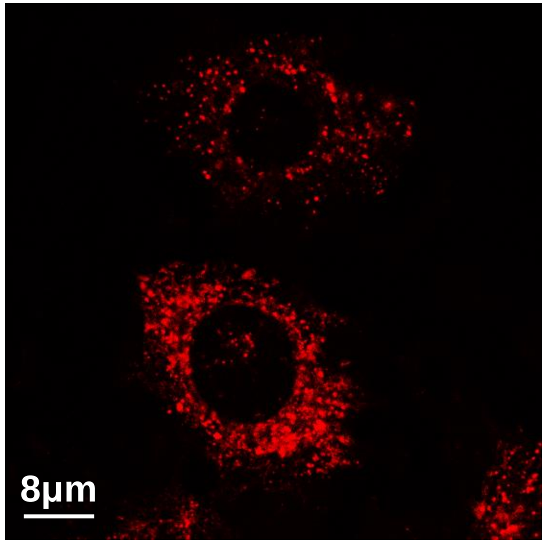

curcumin

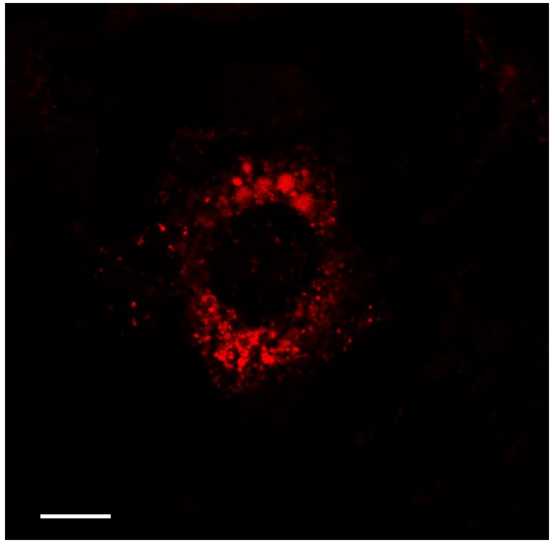

antimycin A/curcumin

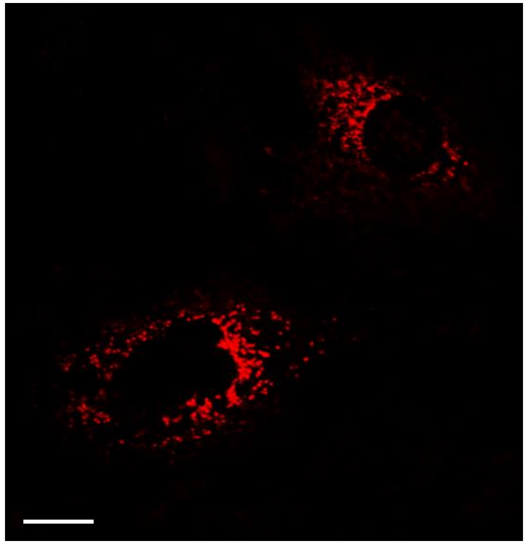

rotenone/curcumin

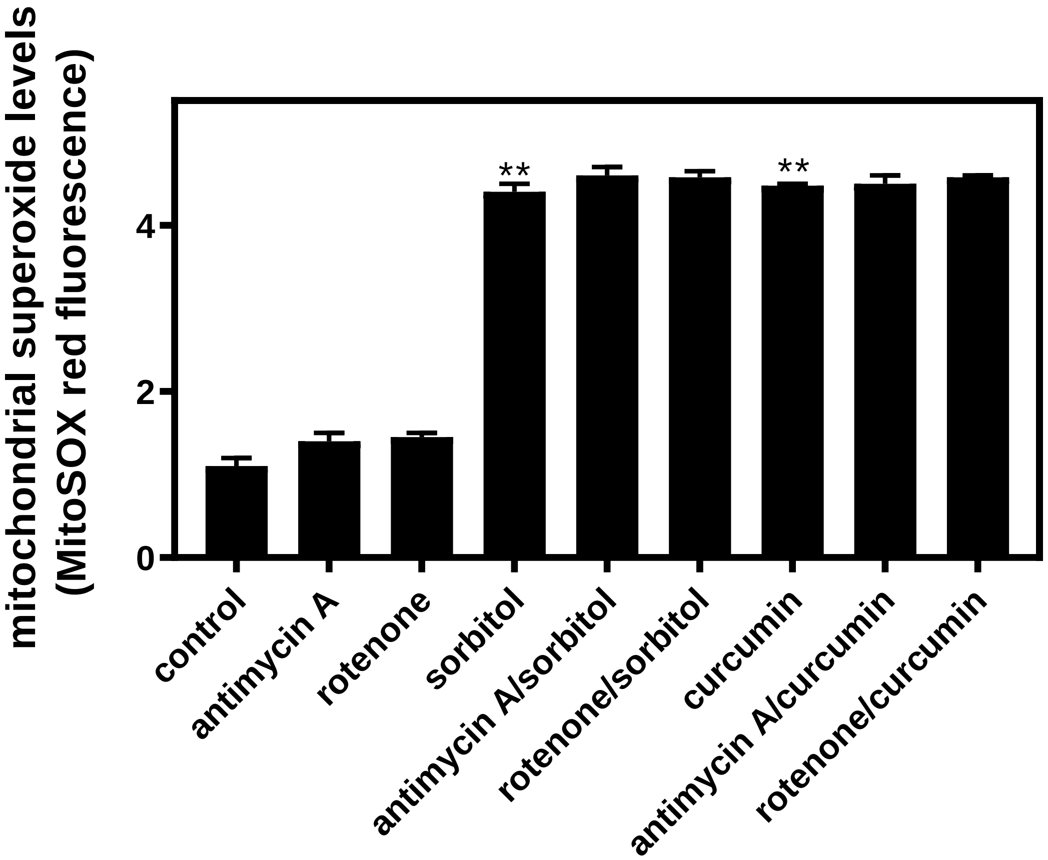

Figure S4 legend   Zikaki et al. (2025)

Figure S4. Neither antimycin A, nor rotenone interfere with sorbitol- or curcumin-induced mitochondrial superoxide generation in H9c2 cardiac cells. H9c2 cells were grown in ibidi μ-Slides and were left untreated (control), exposed to antimycin A, rotenone, sorbitol, curcumin, or pretreated with either of the inhibitors followed by sorbitol or curcumin, in the presence of the respective inhibitor. Cells were next labelled with MitoSOX red CMXRos (5 μM in serum-free DMEM) for 10 min at 37˚C. After washing them in PBS, DMEM was added in the wells and cells were visualized under a Zeiss Axioplan microscope. Fluorescence intensity was quantified by scanning densitometry and plotted (respective graph). Results are means ± SEM for at least three independent experiments. ** $p < 0.001$ compared to control values.
